# Reevaluation of the Impact of the Novel Likely Pathogenic Variant c.1286_1288delAGA in the *ATP8A2* Gene: A 7‐Year Follow‐Up With Clinical, Genetic, and ACMG Insights in an Iranian Family

**DOI:** 10.1002/mgg3.70081

**Published:** 2025-02-11

**Authors:** Samira Kalayinia, Hamed Hesami, Reza Shervin Badv, Maryam Rabbani, Zahra Rezaei, Zohreh Hosseinkhani, Sedighe Nikbakht, Ameneh Sharifi, Bahman Akbari, Siamak Mirab Samiee, Nejat Mahdieh

**Affiliations:** ^1^ Cardiogenetic Research Center Rajaie Cardiovascular Institute Tehran Iran; ^2^ Rajaie Cardiovascular Institute Tehran Iran; ^3^ Children's Medical Centre, Pediatrics Center of Excellence Tehran University of Medical Sciences Tehran Iran; ^4^ Department of Prosthodontics, School of Dentistry Shahid Beheshti University of Medical Sciences Tehran Iran; ^5^ Pediatric Neurology Department, Pediatrics Center of Excellence, Children's Medical Center Tehran University of Medical Sciences Tehran Iran; ^6^ Growth and Development Research Center Tehran University of Medical Sciences Tehran Iran; ^7^ Sleep Disorders Research Center Kermanshah University of Medical Sciences Kermanshah Iran; ^8^ Fetal and Pediatric Cardiovascular Research Center, Children's Medical Center Tehran University of Medical Sciences Tehran Iran

**Keywords:** *ATP8A2* gene, CAMRQ syndrome, cerebellar ataxia, mental retardation

## Abstract

**Background:**

Cerebellar ataxia, mental retardation, and dysequilibrium (CAMRQ) syndrome is a rare neurodevelopmental disorder characterized by non‐progressive cerebellar ataxia, intellectual disability, and cerebellar atrophy. Despite its rarity, CAMRQ syndrome poses significant challenges due to its heterogeneous genetic etiology and complex clinical presentation. This study details the evolving clinical phenotype over 7 years in a male with CAMRQ4 syndrome caused by an in‐frame deletion variant in *ATP8A2* gene.

**Methods:**

A detailed clinical evaluation was performed, accompanied by tests and imaging studies. Clinical and genetic investigations, including segregation analysis, were carried out to confirm the pathogenicity of the identified variant. The evolving clinical phenotype of the patient, including developmental delay, cerebellar ataxia, and hand‐foot crawling, was thoroughly investigated.

**Results:**

A 10‐year‐old male patient with CAMRQ syndrome exhibited typical clinical manifestations including impaired motor coordination, cognitive impairment, and balance disturbances. Genetic analysis revealed a homozygous in‐frame deletion variant (c.1286_1288delAGA) in the *ATP8A2* gene, implicating *ATP8A2* in the pathogenesis of CAMRQ syndrome. This variant was predicted to be likely pathogenic and deleterious, in accordance with its segregation in affected family members. Our findings expand the mutational spectrum of ATP8A2‐associated CAMRQ syndrome and underscore the importance of comprehensive genetic testing in diagnosing rare neurological disorders.

**Conclusion:**

The identification of an in‐frame deletion variant in the *ATP8A2* gene enhances our understanding of CAMRQ syndrome and highlights the phenotypic variability of the disorder. Our study contributes to the elucidation of CAMRQ syndrome by identifying a novel genetic variant and elucidating its clinical and genetic implications. Further research is warranted to advance our understanding of CAMRQ syndrome and to improve patient care and management strategies.

## Graphical abstract

This study reports a 7‐year follow‐up of a male with CAMRQ4 syndrome caused by a homozygous in‐frame deletion (c.1286_1288delAGA) in *ATP8A2*. Clinical and genetic analyses confirm its pathogenicity, expanding the mutational spectrum of *ATP8A2*‐related CAMRQ and underscoring the importance of comprehensive genetic testing in rare neurological disorders.

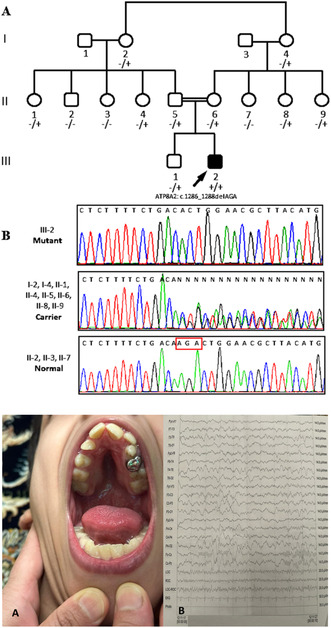

## 1 Introduction

Cerebellar ataxia, mental retardation, and dysequilibrium (CAMRQ) syndrome is defined by non‐progressive cerebellar ataxia, intellectual disability, and cerebellar atrophy (Fadeel and Xue 2009); the clinical manifestations of CAMRQ syndrome encompass a spectrum of neurological deficits, including impaired motor coordination, seizures, dysarthria, optic atrophy, severe hypotonia, cognitive impairment, and balance disturbances such as quadrupedal gait (Alsahli et al. 2018; Martín‐Hernández et al. 2016). Despite its extremely rare occurrence, this syndrome represents a significant challenge for both clinicians and researchers due to its intricate clinical presentation and heterogeneous genetic etiology.

CAMRQ syndrome can arise from pathogenic variants in several genes including the very low‐density lipoprotein receptor (*VLDLR*) (CAMRQ1; MIM: 224050), WD repeat domain 81 (*WDR81*) (CAMRQ2; MIM: 610185), carbonic anhydrase 8 (*CA8*) (CAMRQ3; MIM: 613227) and ATP8A2 designated as CAMRQ4 (MIM: 615268) (Alsahli et al. 2018). Recessive variants of ATP8A2 are associated with CAMRQ type 4 (Onat et al. 2013). This gene plays a critical role in the transport of aminophospholipids into the cytoplasmic leaflet in tissues expressing it, including the cerebrum, cerebellum, spinal cord, retina, and testes (Cacciagli et al. 2010; Coleman et al. 2009; Zhu et al. 2012).

To date, CAMRQ syndrome has been documented in over 50 patients worldwide, highlighting its significant global impact. More than 50 variants have been implicated in the etiology of CAMRQ, with a notable prevalence of missense and nonsense variants in the *ATP8A2* gene. Approximately 7% of these variants are frameshift mutations, highlighting the genetic diversity contributing to the syndrome. It is important to recognize that certain diseases, including CAMRQ, may exhibit ethnic‐specific frequencies, reflecting the genetic heterogeneity among different populations (Cengiz et al. 2017; Mahdieh et al. 2016). Understanding these patterns can aid in more accurate diagnosis and personalized management of CAMRQ syndrome in diverse ethnic groups. This knowledge emphasizes the need for continued research and comprehensive genetic screening to identify and characterize the full spectrum of *ATP8A2* mutations and their clinical correlations.

Notably, a homozygous missense variant (c.1339G>A, p.Gly447Arg) in the ATP8A2 gene was previously identified in an Iranian family. In this study, we present a case initially reported 7 years ago with limited clinical and genetic data (Saghazadeh et al. 2018). In this study, we aim to reevaluate the case by incorporating 7 years of clinical follow‐up, family genetic analysis, biochemical findings, segregation studies, and ACMG criteria to provide a comprehensive understanding of the variant's impact. Bioinformatic analyses, along with clinical and genetic investigations, including segregation analysis, confirmed the pathogenicity of this mutation. Our findings highlight the importance of comprehensive genetic analyses in elucidating the underlying molecular mechanisms of CAMRQ and furthering our understanding of its pathogenesis.

## 2 Methods

### 2.1 Ethical Compliance

Every participant gave their informed consent, and the study procedure was approved by the ethics committee of the Rajaie Cardiovascular Medical and Research Center. This study adhered to ethical principles outlined in the Declaration of Helsinki.

### 2.2 Clinical Investigations

**FIGURE 1:**
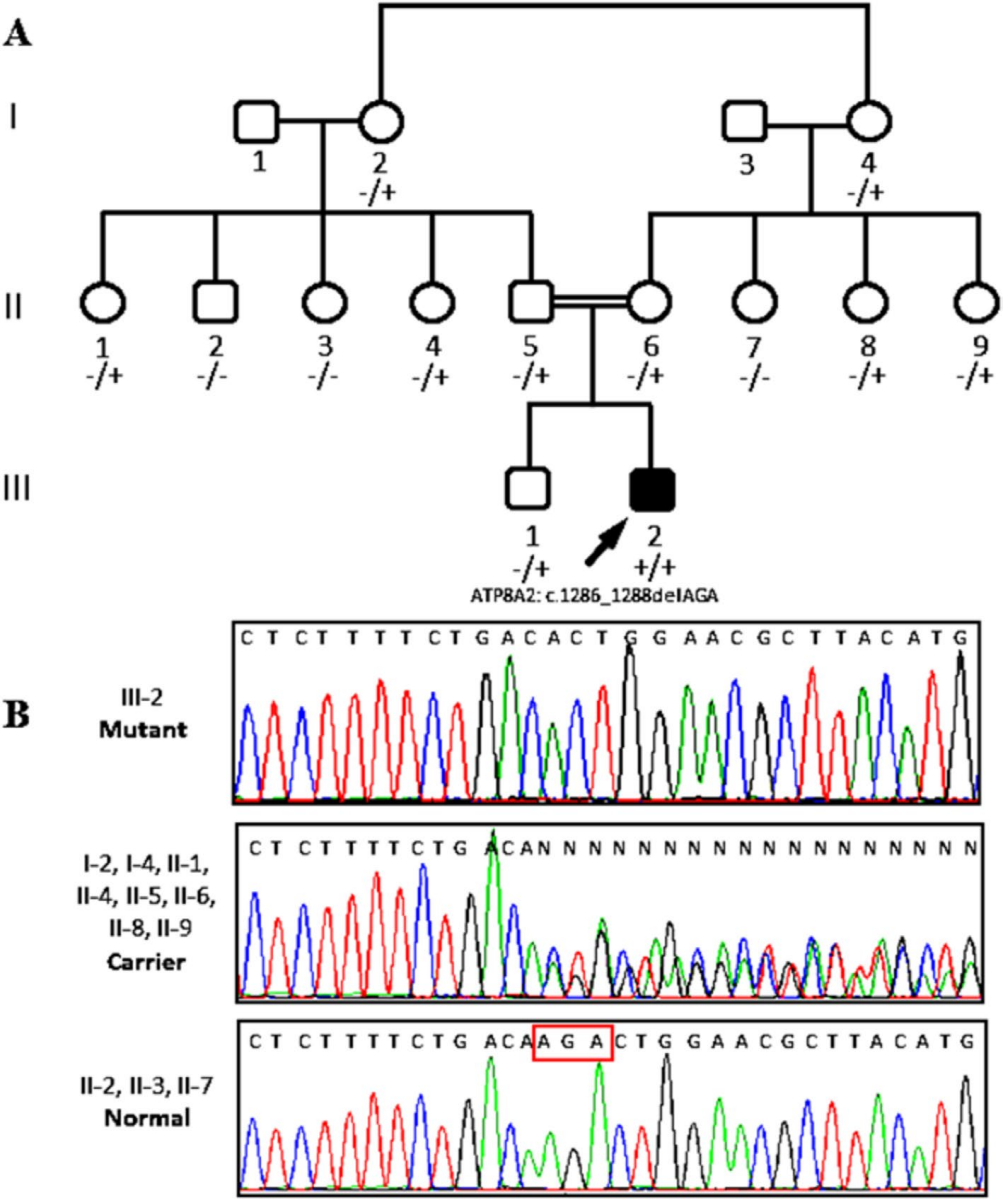
(A) Family pedigree of the patient. (B) Electropherogram of DNA sequencing of the patient, carriers, and normal individuals in the family.

A 10‐year‐old male with a history of developmental delay presented to our clinic accompanied by his family. He was the second child in a family of four individuals, born to consanguineous parents (cousins). The parents and his younger brother showed no signs of illness or abnormalities. At the time of birth, he exhibited an anterior open bite, a high vaulted and constricted maxillary arch, and missing lateral incisors (Figure 1A). By 4 months of age, he was severely hypotonic and as he grew up, signs of cerebellar ataxia became evident; he experienced persistent difficulties with balance and coordination since childhood. The patient also has cognitive impairments, classified as moderate intellectual disability.

Remarkably, he has adapted to using hand‐foot crawling for short distances since early adolescence. During a comprehensive neurological evaluation, he displayed a wide‐based, unsteady gait with frequent falls, requiring support for bipedal ambulation. When unaided, the patient preferred to move using his hands and feet, exhibiting a gait pattern reminiscent of quadrupedalism. The neurological examination revealed dysmetria, intention tremor, and dysdiadochokinesia. Cognitive assessments were consistent with his previous diagnosis of intellectual disability.

Given these neurological findings, we conducted further advanced diagnostic assessments, including an electroencephalogram (EEG), electromyography, and a comprehensive metabolic examination (Figure 1B). Brain magnetic resonance imaging (MRI) was performed using axial T1, T2 flair, and sagittal T2 sequences without contrast media. The patient's serum CK levels were measured at 109 U/L. A biopsy of the left vastus lateralis muscle was also conducted, revealing no significant histochemical pathological findings. With most of the diagnostic findings being normal, the patient was referred for genetic study.

### 2.3 Sample Collection and DNA Extraction

Peripheral blood specimens from all available pedigree members—either affected or healthy—were used to extract genomic DNA according to standard protocols. Quality control assessments were conducted to ascertain the integrity and purity of the extracted DNA, employing NanoDrop (NanoDrop 2000 Spectrophotometers, The Thermo Scientific) and gel electrophoresis.

### 2.4 Variant Annotation and Prioritization

Variant was annotated using widely recognized databases such as dbSNP, ClinVar, and the Exome Aggregation Consortium (ExAC), providing comprehensive functional annotations. Functional prediction of identified variants was conducted using multiple tools, including MutationTaster and CADD, following the American College of Medical Genetics and Genomics (ACMG) variant interpretation criteria (Richards et al. 2015). Additionally, variant was prioritized based on their potential pathogenicity and relevance to the study phenotype.

### 2.5 Segregation Analysis and Sanger Sequencing Validation

The identified variant underwent Sanger sequencing as the gold standard for validation. PCR primers were designed flanking the genomic region harboring the variant, ensuring specificity and optimal amplification conditions. Thirty‐five cycles of denaturing at 95°C for 30 s, annealing at 60°C for 30 s, and extension at 72°C for 30 s comprised the PCR conditions. Sanger sequencing was performed by ABI3500 (Applied Biosystems, USA) platform. Validation was deemed successful if the Sanger sequencing results confirmed the presence of the variant, with a focus on obtaining high‐quality chromatograms and unambiguous electropherograms.

## 3 Results

### 3.1 Clinical Findings

In this clinical case, a 10‐year‐old male patient with a history of developmental delay and consanguinity in the family was assessed for his unique presentation of motor and cognitive impairments. As previously reported (Saghazadeh et al. 2018), the patient was born by cesarean section, with normal weight, length, and head circumference, and his Apgar score was within the expected range. Hypotonia was first detected during a routine immunization. At the age of 3 months, the patient developed severe pneumonia, requiring ICU care. After recovery, he was referred to a neurology clinic due to his inability to maintain head control. Neonatal screening tests, including hemoglobin, TSH, and metabolic screenings, were all normal. Despite undergoing a comprehensive neurorehabilitation program from 4 months to 1.5 years, there was no significant improvement in his head lag. By 9 months, additional clinical and paraclinical evaluations were initiated due to persistent symptoms such as severe hypotonia, developmental delay, speech problems, strabismus, and a high‐arched palate. The patient was unable to sit, stand, or walk independently. Electromyography and sensory nerve conduction studies revealed no evidence of lower motor neuron damage. A muscle biopsy showed a slight predominance of type I fibers, and MRI scans of the brain at 5 months and 2 years showed no abnormalities.

**FIGURE 2:**
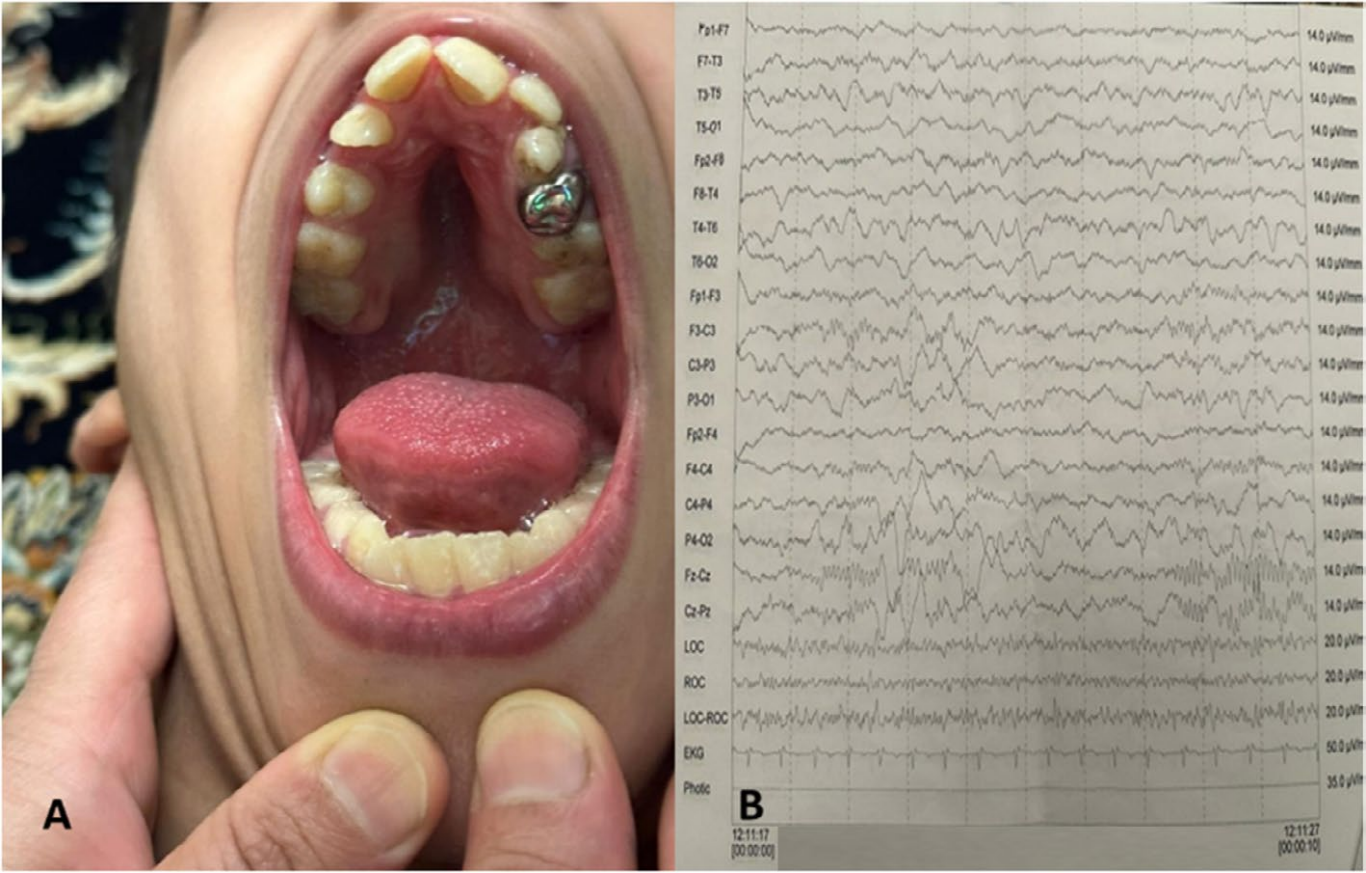
(A) The image shows an anterior open bite, with a high vault and constricted maxillary arch missing lateral incisors in our patient. (B) The image shows normal EEG which was conducted to assess brain function.

At age of 9 years, despite displaying an unusual gait and cognitive challenges, most diagnostic assessments yielded normal results (Figure 2A). Neurological examination confirmed the presence of cerebellar ataxia, evidenced by a wide‐based, unsteady gait, dysmetria, intention tremor, and dysdiadochokinesia. Cognitive assessment was in line with the patient's prior diagnosis of moderate mental retardation. Despite these findings, advanced neurological assessments, including EEG and Electromyography, did not identify any specific abnormalities (Figure 2B). A comprehensive metabolic examination did not reveal any significant metabolic disorders that might explain the patient's symptoms. Additionally, brain MRI scans, which included axial T1, T2 FLAIR, and sagittal T2 sequences without contrast media, were normal, showing no structural abnormalities that could account for the patient's condition. An anomaly was found in the elevated serum CK levels, which were measured at 109 U/L, suggesting the possibility of a muscle disorder. However, subsequent muscle biopsy of the left vastus lateralis did not reveal any significant histochemical pathology, contradicting the serum CK findings and leaving the etiology of the elevated levels unexplained. Given the largely unremarkable findings from the extensive clinical investigations, the patient was referred for genetic testing to explore the potential for an underlying genetic condition that might elucidate the etiology of his clinical presentation. The referral for genetic study was considered the next rational step given the consanguinity of the parents and the absence of a clear diagnosis despite thorough assessment. This would potentially provide insights into rare genetic mutations or syndromes that could manifest with the observed symptoms, especially considering the patient's adaptation to hand‐foot crawling and other neurological signs without a clear structural or metabolic cause (Table 1).

**TABLE 1:** Comparison of clinical and genetic findings between the initial and updated report.

Aspect	Initial report	Updated report
Age	3 years old at the time of initial assessment	10 years old at the time of reassessment
Clinical signs	Severe hypotonia, global developmental delay, speech delay, strabismus, and inability to sit, stand, or walk	Predominantly cerebellar ataxia with dysmetria, intention tremor, and dysdiadochokinesia
Cognitive function	Global developmental delay noted	Moderate mental retardation confirmed by cognitive assessment
Additional symptoms	Strabismus and high‐arched palate	Wide‐based gait and adaptation to hand‐foot crawling
Biochemical findings	Normal screening tests for metabolic conditions	Elevated serum CK levels (109 U/L), though muscle biopsy revealed no significant pathology
Diagnostic techniques	Normal EMG and nerve conduction studies; muscle biopsy with mild Type I fiber predominance	EMG and EEG were normal; muscle biopsy findings were unremarkable
Focus on genetics	Case highlights the identification of the ATP8A2 variant but lacks detailed ACMG classification and segregation analysis	The variant was heterozygote or normal in all healthy members of the family. Detailed ACMG classification (PM2, PM3, PM4, PM5, PP3, PP5) supports pathogenicity

### 3.2 Genetic Findings

To understand the inheritance pattern of the identified c.1286_1288delAGA (p.Lys429del), within exon 14 of the *ATP8A2* gene (NM_016529.6), it was confirmed in the patient as a homozygote form. Parents and his brother were carrier, heterozygote form. Other healthy members of the pedigree had normal sequence in this position or heterozygous (Figure 1). MutationTaster classifies the variant as disease‐causing with a high level of confidence. This prediction aligns with the homozygous presentation of the variant in affected proband and supports its potential pathogenic role in the context of *ATP8A2*‐related CAMRQ disorder. CADD assigns a high deleteriousness score to the c.1286_1288delAGA variant (CADD phred > 20). This high score suggests that the variant is likely to have a significant impact on protein function and may contribute to the observed CAMRQ phenotypes in the patient. In accordance with the ACMG guidelines, the c.1286_1288delAGA variant meets multiple criteria, PM2 (Pathogenic Moderate: Extremely low frequency), PM4 (Pathogenic Moderate: Protein length changes), PM5 (Pathogenic Moderate: Different amino acid change as a known pathogenic variant), PP3 (Pathogenic Supporting: computational prediction), and PM3 (detected in trans with a likely pathogenic variant) (https://clinicalgenome.org/working‐groups/sequence‐variant‐interpretation/), supporting its classification as a likely pathogenic variant.

## 4 Discussion

Our study contributes to the growing body of knowledge on CAMRQ syndrome by expanding on the clinical and genetic aspects of a novel in‐frame deletion variant in the *ATP8A2* gene. Building upon a previously reported case (Saghazadeh et al. 2018), our current study provides new insights through extended clinical follow‐up and advanced genetic analyses. These findings further support the pathogenicity of the *ATP8A2* variant and its role in CAMRQ syndrome.

The clinical manifestations of CAMRQ syndrome observed in our patient are consistent with previously reported cases, characterized by non‐progressive cerebellar ataxia, intellectual disability, and balance disturbances (Alsahli et al. 2018). Notably, our patient exhibited unique features, including adaptation to hand‐foot crawling, which underscores the phenotypic variability inherent in CAMRQ syndrome (Alsahli et al. 2018; Zhu et al. 2012). The absence of structural abnormalities on brain MRI highlights the challenge of diagnosing CAMRQ syndrome, as traditional imaging modalities may not always capture the underlying neurological pathology associated with this disorder (Aderinto et al. 2023).

Genetic analysis revealed a homozygous likely pathogenic frameshift variant (c.1286_1288delAGA) in the *ATP8A2* gene, further implicating *ATP8A2* in the pathogenesis of CAMRQ syndrome (Alsahli et al. 2018; Martín‐Hernández et al. 2016; Onat et al. 2013; Cacciagli et al. 2010; Coleman et al. 2009). CAMRQ4 may be more prevalent in our population, similar to other diseases that exhibit ethnic‐specific frequencies. In other words, certain diseases are more commonly found among specific ethnicities. The c.1286_1288delAGA variant was predicted to be disease‐causing and deleterious, in line with its segregation in affected family members and its absence in healthy individuals; it was classified as a likely pathogenic variant according to ACMG guideline (Richards et al. 2015). The identification of this variant expands the mutational spectrum of ATP8A2‐associated CAMRQ syndrome and underscores the importance of comprehensive genetic testing in diagnosing rare neurological disorders (Heshmatzad et al. 2021; Mahdieh et al. 2024). This variant may be common in Iran and have arisen from a founder mutation, similar to variants in *CYP21A* and *SLC26A4* genes observed in Iranian and Turkish populations (Cengiz et al. 2017; Rabbani et al. 2011). Additionally, it may be attributed to a mutational hotspot; however, further studies are required to confirm this hypothesis.

Our findings underscore the critical role of patient follow‐up in confirming the pathogenicity of genetic variants underlying neurodevelopmental disorders like CAMRQ syndrome. Exome sequencing offers a powerful tool for elucidating the genetic basis of heterogeneous disorders and guiding personalized treatment strategies (Galatolo et al. 2023). Additionally, our study highlights the importance of segregation analysis in confirming the pathogenicity of identified variants and informing genetic counseling for affected families.

## 5 Conclusion

This study highlights the importance of long‐term follow‐up and comprehensive reanalysis of previously reported cases. The additional clinical and genetic data presented here further validate the likely pathogenic role of the *ATP8A2* variant in CAMRQ syndrome. We believe our study contributes to a deeper understanding of CAMRQ syndrome by elucidating its clinical and genetic implications. Our findings suggest that CAMRQ4 disorder may be more prevalent in the Iranian population, potentially due to a founder mutation or a mutational hotspot. The presented family demonstrates the clinical and genetic complexities of cerebellar ataxia, mental retardation, and disequilibrium syndrome, contributing to the global knowledge base on this disorder. Further research is essential to elucidate the molecular mechanisms underlying CAMRQ syndrome, develop targeted therapeutic strategies, and explore the broader implications of *ATP8A2* mutations in diverse populations. Such efforts could pave the way for improved diagnostic approaches and interventions aimed at mitigating the impact of this debilitating condition.

## Author Contributions

S.K. and N.M. drafted the manuscript. R.S.B., Z.R., S.N. and M.R. collected and interpreted patient's clinical data. H.H., S.K., S.M.S., B.A., A.S. and N.M. reviewed and revised the manuscript. S.K., Z.H. and N.M. performed NGS data analysis. N.M. and S.K. designed the study. All authors contributed to the study's conception and design.

## Ethics Statement

The study complies with the Declaration of Helsinki. Ethical approval was obtained from the Ethics Committees of Rajaie Cardiovascular Medical and Research Center, Iran University of Medical Sciences, Tehran, Iran (IR.RHC.REC.1403.033).

## Consent

Written informed consent was obtained from the participants.

## Conflicts of Interest

The authors declare no conflicts of interest.

## Data Availability

Data sharing not applicable to this article as no datasets were generated or analysed during the current study.
